# Smart window films with ultra-broadband radiative regulation for all-season building energy saving

**DOI:** 10.1039/d6ra04939g

**Published:** 2026-08-18

**Authors:** Wenjuan Jiang, Haining Ji, Juantao Zhang, Xiyu Wu, Runteng Luo, Zhi Zeng, Tianjian Xiao

**Affiliations:** a School of Physics and Optoelectronics, Xiangtan University Xiangtan Hunan 411105 P. R. China sdytjhn@126.com

## Abstract

Windows are critical pathways for heat exchange and thermal radiation transfer across building envelopes, making temperature-adaptive spectral control essential for year-round building energy conservation. However, the strong coupling among visible, solar, and thermal-infrared responses, together with the high structural sensitivity and complex design space of multilayer films, makes coordinated multi-objective optimization challenging. To address this issue, this study proposes a Deep Q-network (DQN)-based reinforcement-learning inverse design framework coupled with the transfer matrix method (TMM). A two-stage optimization strategy is employed: dielectric materials and layer thicknesses are first optimized simultaneously using a symmetric dielectric/VO_2_/dielectric cavity, after which the selected material system is fixed to optimize multilayer structures with different layer numbers. The resulting CaF_2_/VO_2_ alternating symmetric system enables synergistic regulation across the solar and mid-to-far-infrared regions, with the five-layer CaF_2_/VO_2_/CaF_2_/VO_2_/CaF_2_ structure exhibiting the best overall performance. It achieves visible transmittances of 51.06% and 46.97% in the low- and high-temperature states, respectively, together with a solar modulation capability of 12.28% and an average emissivity modulation of 47.33% over 2.5–25 µm. Electric-field analysis attributes this multispectral response to the temperature-dependent reconstruction of optical interference and electromagnetic coupling induced by the VO_2_ phase transition, while angular analysis confirms stable temperature-selective behavior over a broad range of incident angles. EnergyPlus simulations further demonstrate energy-saving potential across different climate zones, with a maximum annual energy saving of 644.21 MJ per m^2^ per year in the hot semi-arid climate zone (BSh). These results demonstrate that the proposed DQN-assisted framework provides an effective strategy for designing temperature-adaptive smart windows with coordinated solar and ultra-broadband thermal-radiation regulation.

## Abstract 2

Inverse-designed CaF^2^/VO^2^ multilayers enable temperature-adaptive regulation of solar and mid-to-far-infrared radiation for climate-responsive, all-season building energy savings.

## Graphical abstract

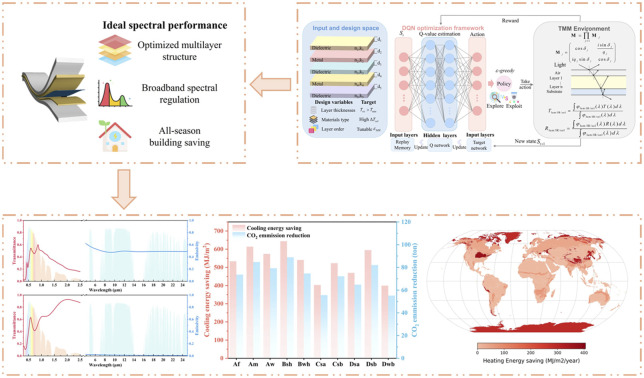

## 1 Introduction

Against the backdrop of the ongoing global low-carbon transition, reducing building operational energy consumption is of great significance for achieving carbon emission reduction targets.^1–3^ As the primary pathway for solar radiation entry and heat exchange across building envelopes, managing thermal radiation through windows is an effective way to improve thermal comfort and reduce energy consumption.^4,5^ Although traditional static energy-saving glass can reduce some of the thermal load, its fixed spectral response struggles to adapt to the conflicting heating and cooling control requirements that arise with seasonal changes, and may even result in additional energy consumption.^6^ Consequently, the development of smart window materials capable of adaptively modulating their light and heat transmission in response to ambient temperature has become a research focus in building energy efficiency.^7–10^ This requires that window materials precisely match the building's solar heat gain and heat radiation exchange needs across different seasons, driving the collaborative design and continuous optimization of phase-change materials and multi-layer optical structures.^6,11,12^ However, designing energy-efficient windows that combine multi-objective coordinated optimisations, manufacturability, and cost advantages still poses numerous challenges.^13–15^ The development of metamaterials has opened up new photonic avenues for overcoming this bottleneck. By using subwavelength structures or multilayer resonant cavities to directionally control light propagation, phase matching, and radiative coupling, metamaterials demonstrate unique advantages in temperature-adaptive photothermal management.^16–20^ However, current metamaterial design often relies on empirical parameter sweeps or iterative experimentation. When the scope expands to include simultaneous multi-objective optimization, traditional forward design methods are limited by their limited exploration efficiency in high-dimensional parameter spaces, making it difficult to efficiently obtain optimal structures that satisfy multi-objective constraints.^21–24^

In the field of reverse engineering for complex optical structures, researchers have begun to incorporate machine learning methods to reduce reliance on human expertise and point-by-point parameter sweeping, thereby improving the efficiency of materials development. For instance, Jiang and Fan introduced ResNet generative neural networks into the global optimization of photonic structures, enabling the simultaneous search for structural parameters and material types;^25^ Lininger *et al.* applied convolutional neural networks to the inverse design of layered thin-film materials to address the challenges posed by the vast parameter space of multilayer structures and the low efficiency of traditional search methods;^26^ Han *et al.* integrated the transfer matrix method into a backpropagation network to enhance the efficiency of optimizing multilayer film thickness.^27^ These studies demonstrate that data-driven methods can accelerate the design of complex spectra and, to some extent, overcome the efficiency limitations of traditional parameter scanning.^28–30^ However, such methods typically rely on pre-generated datasets, fixed structural encodings, or explicit target spectral mappings. When design tasks extend to multifunctional, multi-constrained dynamic spectral design objectives, the non-uniqueness of the relationship between structure and performance, as well as the trade-offs among objectives, significantly increase the difficulty of optimization.^23,31^ Therefore, there is an urgent need to develop a multi-objective machine learning method coupled with a fast physical solver, enabling ultra-broadband multi-objective co-optimization of multilayer smart windows while ensuring basic daylighting performance.^32–34^

Based on this, this paper proposes a Deep Q-network (DQN)-based reinforcement learning inverse design strategy for multilayer smart windows, enabling automatic parameter search of multilayer film structures and the simultaneous optimization of ultra-wideband multispectral performance. This proposed method uses D/M/D-type resonant cavities as the basis for structural design, rapidly obtains spectral responses at high and low temperatures *via* the transfer matrix method, and establishes a multi-objective evaluation function centered on the regulation of visible light transmittance, solar modulation, and mid-to-far-infrared emissivity. During continuous interactions, the DQN agent dynamically adjusts layer thickness parameters based on spectral feedback, progressively learning a design policy that maps structural states to optimal actions, thereby avoiding the reliance on empirical configurations and large-scale parameter sweeps inherent in traditional forward design. Through this framework, a multilayer film structure integrating visible light transmittance, solar modulation capability, and ultra-broadband thermal radiation management capabilities is obtained. By further integrating electromagnetic field analysis, angle-dependent response, and building energy consumption simulation, the study elucidates the underlying multispectral synergistic regulation mechanism and practical energy-saving potential. This work provides a new methodological approach for the intelligent design of phase-change smart windows and their application in building energy conservation.

## 2 Methodology

### 2.1 The design principles of smart windows

**Fig. 1:**
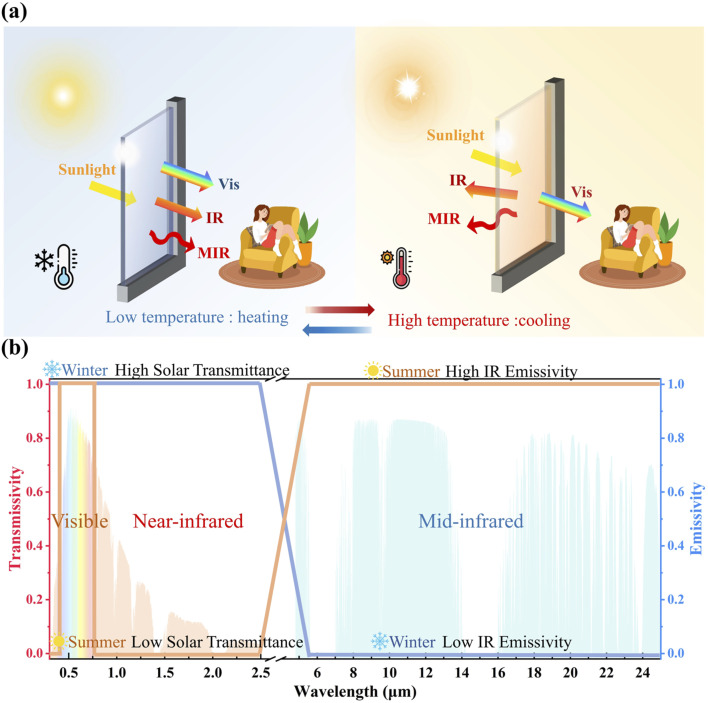
Design principles and spectral control requirements for an ideal smart window. (a) Energy control mechanisms of smart windows under low- and high-temperature conditions; (b) schematic diagram of the spectral response of an ideal smart window in the solar and mid-to-far-infrared bands.

Thermal radiation can be divided into the visible light band (0.38–0.78 µm), the near-infrared band (0.78–2.5 µm), and the mid- and far-infrared bands (2.5–25 µm).^7^ For building windows, the visible light spectrum primarily determines the window's transparency and indoor visual comfort; the near-infrared spectrum, which carries a large proportion of solar heat, is a major source of solar heat gain indoors; and the mid- and far-infrared spectra, which constitute the primary range of thermal radiation emitted by objects at room temperature, dominate the process of radiative heat exchange between the window and the outdoor environment.^11^ The operating principle of an ideal energy-efficient window is shown in Fig. 1(a). To satisfy building daylighting requirements, a window must maintain high visible transmittance under all temperature conditions. For energy-saving performance, it should exhibit opposing temperature-dependent spectral responses in the near-infrared and mid-to-far-infrared bands. Specifically, under cold conditions, windows should have high near-infrared transmittance to enhance solar heat gain, while maintaining low mid-to-far-infrared emissivity to reduce indoor heat radiation loss; conversely, under high-temperature conditions, windows should have low near-infrared transmittance to suppress solar heat input, while possessing high mid-to-far-infrared emissivity to enhance heat radiation release.^35,36^ The corresponding ideal spectral characteristics are shown in Fig. 1(b). The coordinated control of solar radiation and mid-to-far-infrared bands is key to achieving year-round energy-saving optimization for windows.^37^

### 2.2 Design and modelling

In the specific modeling process, this paper first starts from the spectral control mechanism and models the multilayer thin-film structure as a D/M/D-type resonator. Previous studies have shown that such structures can simultaneously utilize the reflective properties of the metal layers toward near-infrared radiation, as well as the anti-reflective, phase-compensating, and cavity-interference modulation effects of the dielectric layers. The positions of spectral peaks and valleys, as well as the intensity of modulation, are highly sensitive to the thickness of the cavity layers, providing a sound physical basis for achieving precise spectral shaping through layer thickness adjustment.^38,39^

Based on this, this paper has established a library of candidate materials that includes metallic materials, dielectric materials, high-refractive-index non-metallic materials, and functional materials. Among these, metallic materials are primarily used to enhance reflectivity in the near-infrared spectrum,^40^ such as Ag and Al. Dielectrics and materials with high refractive indices are primarily used to reduce reflection losses in the visible light spectrum and to achieve precise control over the position and intensity of spectral lines by adjusting the equivalent optical thickness and the resonant conditions within the cavity,^41^ such as AlN, Al_2_O_3_, CaF_2_, HfO_2_, *etc.* Functional materials enable the film system to dynamically modulate its optical properties through temperature-dependent changes in their optical constants.^42^ In this study, VO_2_ was selected as the functional material. The optical constants of all candidate materials were obtained from the literature, and their sources are provided in Table S1 of the SI. Although intrinsic VO_2_ exhibits a transition temperature of approximately 68 °C, chemical doping and strain engineering provide effective routes for shifting the transition temperature toward the range suitable for practical building applications. To facilitate algorithm recognition and invocation, this paper further encodes the candidate materials using integer codes. Consequently, any candidate structure can be represented as a combination of a material sequence and a thickness sequence, namely *S* = {*m*_1_,*m*_2_,⋯,*m*_*i*_; *t*_1_,*t*_2_,⋯,*t*_*i*_}. Among them, *m*_*i*_ denotes the material code for the th *i* material layer, and *t*_*i*_ denotes the thickness parameter for the th *i* layer to be optimized.

To achieve the design objectives described in Section 2.1, while maximizing visible light transmittance, solar radiation modulation capability, and thermal radiation management capability, this paper defines the following objective function:1
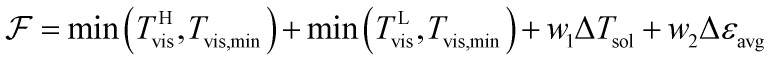
In eqn (1), *T*^H^_vis_ and *T*^L^_vis_ represent the visible light transmittance at high and low temperatures, respectively, and *T*_vis,min_ is the minimum visible light transmittance threshold, set at 0.47 in this paper.^43^ Δ*T*_sol_ denotes solar modulation capability, Δ*ε*_avg_ denotes average emissivity modulation capability, *w*_1_ and *w*_2_ are set to 3 and 1, respectively. This function ensures that basic light transmission requirements are met through threshold constraints, while simultaneously focusing the optimization process on improving solar modulation and infrared emissivity modulation performance.

The light transmittance is calculated using a weighting based on the human eye's photopic visual function and is defined as:2
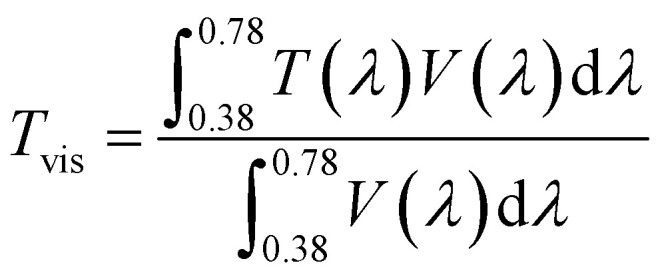
In eqn (2), *T*(*λ*) represents spectral transmittance, and *V*(*λ*) represents the human luminous efficiency function, which reflects the differences in the contribution of different visible wavelengths to the human eye's perception of brightness.

Solar transmittance is calculated using the AM1.5 solar spectrum weighting, and solar modulation capability is defined as the difference in solar transmittance before and after the phase change:3
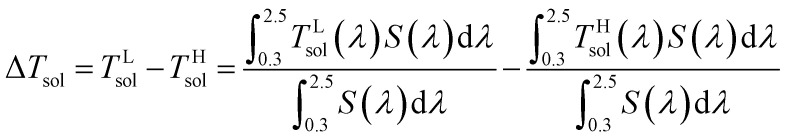


Infrared emissivity is derived from spectral absorptivity. Under the no-scattering approximation, spectral absorptivity can be expressed as:

4 *A*(*λ*) = 1 − *R*(*λ*) − *T*(*λ*)

According to Kirchhoff's law, the spectral emissivity in thermal equilibrium can be approximated as equal to the spectral absorptivity, *i.e. ε*(*λ*) = *A*(*λ*). Since the thermal radiation of objects at room temperature is primarily concentrated in the mid- and far-infrared bands, this paper employs a blackbody radiation-weighted method to calculate the average infrared emissivity. The emissivity modulation capability is defined as follows:5
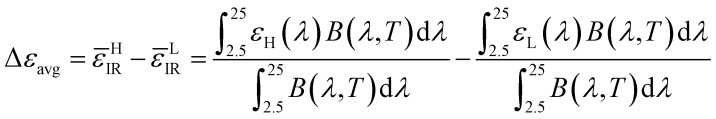
In eqn (5), *B*(*λ*,*T*) represents the spectral radiant intensity of a blackbody at temperature *T*. In this paper, we take *T* = 300 K. According to Planck's law:6
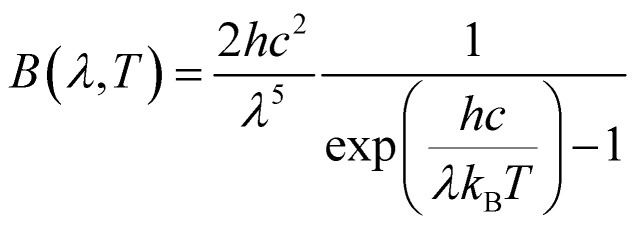
In eqn (6), *h*, *c*, and *k*_*B*_ are the Planck constant, the speed of light in a vacuum, and the Boltzmann constant, respectively.

Optical simulations were performed using COMSOL Multiphysics. A broad-spectrum plane-wave light source was set to incident perpendicularly along the *z*-axis in the model. To characterize the laterally infinite thin-film structure, periodic boundary conditions were applied in the *x* and *y* directions. A perfectly matched layer (PML) was placed along the *z*-axis to absorb the outgoing electromagnetic waves and eliminate boundary reflections. Subsequently, the electric field at a typical wavelength was analyzed to reveal the electromagnetic mechanisms underlying the film's spectral tuning. Furthermore, the spectral response was calculated by varying the incident angle to evaluate the structure's optical stability under different illumination angles.

### 2.3 Core algorithm mechanisms and implementation process

**Fig. 2:**
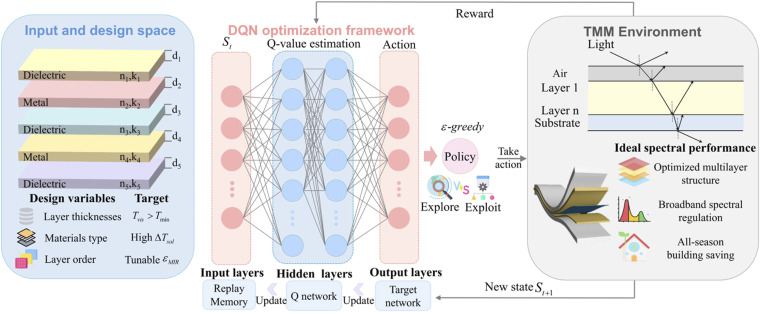
DQN-assisted inverse design workflow for broadband radiative regulation in multilayer smart window films.

Based on the structural parameterization method and performance evaluation function, this paper further constructs a DQN optimization framework to enable the automatic search for multi-layer film thickness parameters. The overall modeling and optimization process is shown in Fig. 2. DQN approximates the action-value function *Q*(*s*,*a*) in reinforcement learning using a neural network, enabling the agent to evaluate the potential contribution of different thickness adjustment actions to future overall performance improvement based on the current state of the film system. Compared to traditional Q-learning, which is limited to low-dimensional discrete state spaces, DQN establishes a nonlinear mapping between states and action values *via* a neural network, rendering it more suitable for complex optimization tasks involving continuous parameters that have been discretized, such as multilayer films. Classic DQN research indicates that experience replay and target networks can effectively mitigate instability issues caused by training sample correlation and non-stationary target values, serving as key mechanisms for enhancing the training stability of deep reinforcement learning.

In the model presented in this paper, to avoid an excessively large combinatorial design space resulting from simultaneous changes in material type, membrane layer thickness, and the number of structural layers, and to investigate the effects of material selection and the number of layers separately, thereby more objectively evaluating the role of the number of layers on the performance of the film system, a two-stage optimization strategy was adopted. In the first stage, based on a three-layer symmetric D/VO_2_/D resonant cavity structure, the environmental state is defined as *S*^(1)^_*t*_ = [*m*_D_, *d*_D_, *d*_VO_2__], where *m*_D_ is the discrete code corresponding to the candidate material, and *d*_D_ and *d*_VO_2__ are the thicknesses of the symmetric D layers on both sides and the intermediate VO_2_ layer, respectively. The action space comprises two types of operations: candidate material switching and layer thickness adjustment, enabling the agent to simultaneously search for combinations of material types and thicknesses. In the second stage, the material system screened in the first stage is fixed and expanded into 3-, 5-, 7-, and 9-layer alternating symmetric structures. At this stage, the environmental state consists solely of the independent layer thickness parameters within the corresponding structure, and the action space is correspondingly simplified to applying discrete increase or decrease operations to a specific independent layer thickness. Since the structure maintains mirror symmetry, an adjustment to a single independent variable will simultaneously affect the film layer at its symmetric position.

Candidate material codes and all independent layer thickness parameters are normalized to the interval [0,1] before being fed into the neural network to minimize the impact of differing numerical scales of variables on the training process. Both stages employ the same thickness adjustment method, namely performing *a* ± Δ*t* operation based on the current layer thickness, where Δ*t* = 0.001, 0.005, or 0.010 µm, enabling the algorithm to balance fine-scale local search with large-scale exploration of the design space. For actions that might cause the layer thickness to exceed the preset upper and lower limits, an action masking mechanism is used to exclude them from the current set of valid actions; in the first stage, repeated selection actions targeting the current material itself are also masked, thereby avoiding invalid state transitions and ensuring that the search process always remains within the preset design space.

During the action selection phase, this paper employs a *ε*-greedy strategy to balance exploration and exploitation. A high random exploration probability is set at the beginning of training to allow the agent to fully explore different layer thickness combinations. As training progresses, *ε* gradually decays, causing the strategy to shift from random exploration to optimal action selection based on Q-value. Specifically, *ε* linearly decays from 1.0 to 0.05 during training, with the decay process covering 90% of the total training steps. In each interaction, the agent selects the valid action with the highest Q-value predicted by the online network with probability 1 − *ε*. This strategy not only prevents the model from getting stuck in local optima too early in training but also ensures that it can effectively leverage the well-learned structural adjustment rules in the later stages of training.^44^

After each action is executed, the environment invokes the transfer matrix method to calculate the updated spectral response of the film system and computes the instantaneous reward based on the comprehensive performance evaluation function 
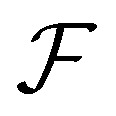
. In this paper, the reward is defined as the difference between the evaluation functions of two consecutive steps:7
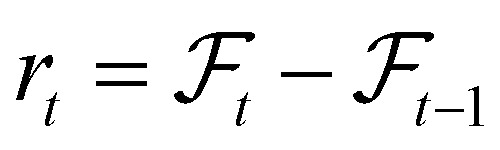


To improve training stability, this paper introduces an experience replay mechanism. During interactions with the environment, the agent stores the state *S*_*t*_, action *a*_*t*_, reward *r*_*t*_, termination flag, and next state *S*_*t*+1_ in an experience pool, and during training, randomly samples small batches of data from the experience pool to update the network parameters. Random sampling reduces temporal correlations between consecutive interaction samples, thereby enhancing the stability of neural network training and improving sample utilization efficiency. Implementation details of the algorithm, including its network structure, design space, initialization, and termination conditions, are summarized in the SI and Table S3.

To test the robustness of the optimization results against random initialization, this paper further conducted three independent training runs on the final five-layer structure selected. The corresponding convergence curves and optimization results are shown in Fig. S2 and Table S4 of the SI, respectively. The cumulative best composite evaluation scores for all three runs increased rapidly during the early stages of training, then gradually stabilized, with final values of 1.7735, 1.7816, and 1.7810, respectively, and an average of 1.7787 ± 0.0045. This indicates that the optimization results exhibit good stability with respect to random initialization.

Table S5 in the SI lists a comparison of the results of this study with those reported in recent studies. The results indicate that the primary advantage of the design proposed in this paper lies in its ability to simultaneously achieve effective solar radiation modulation and significant emissivity switching across a broad wavelength range of 2.5–25 µm, thereby enabling balanced multispectral regulation beyond the conventional 8–13 µm atmospheric window. Unlike commercial static low-emissivity (low-E) glass, this design also enables temperature-adaptive regulation of solar radiation transmittance and thermal radiation.

## 3 Results and discussion

### 3.1 Analysis of optical properties

**Fig. 3:**
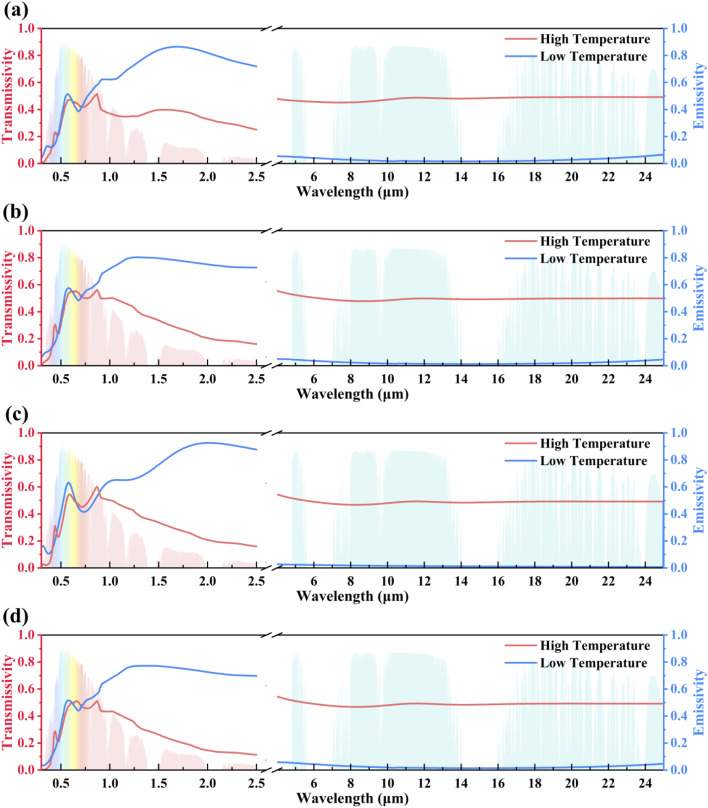
Spectral response of CaF_2_/VO_2_ alternating symmetric multilayer structures with different numbers of layers. (a) Three-layer film; (b) five-layer film; (c) seven-layer film; (d) nine-layer film.

The optimization results from the first stage indicate that the CaF_2_/VO_2_/CaF_2_ structure achieved the best overall spectral tuning performance among the candidate structures evaluated. The optimization results and spectral curves for the other candidate material systems are presented in Table S2 and Fig. S1, respectively. Therefore, CaF_2_/VO_2_ was selected as the material system for subsequent structural expansion. In the second stage, the independent layer thickness parameters were optimized for 3-, 5-, 7-, and 9-layer CaF_2_/VO_2_ alternating symmetric structures. Table 1 summarizes the layer thickness parameters and corresponding optical properties of the optimized structures with different numbers of layers; the spectral transmittance in the solar band and the emissivity in the mid- and far-infrared regions are shown in Fig. 3.

**Table 1:** Comparison of optimized structures and optical performance^a^

Structure (thickness/nm)	*T*_vis,l_	*T*_vis,h_	Δ*T*_sol_	Δ*ε*_avg_	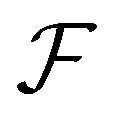
CaF_2_(282.33)/VO_2_(39.71)/CaF_2_(282.33)	45.46%	41.71%	12.69%	45.21%	1.705
CaF_2_(86.51)/VO_2_(16.34)/CaF_2_(207.32)/VO_2_(16.34)/CaF_2_(86.51)	51.06%	46.97%	12.28%	47.33%	1.781
CaF_2_(93.49)/VO_2_(10)/CaF_2_(202.14)/VO_2_(12)/CaF_2_(202.14)/VO_2_(10)/CaF_2_(93.49)	50.09%	45.94%	12.43%	47.90%	1.781
CaF_2_(86.94)/VO_2_(10)/CaF_2_(10)/VO_2_(10)/CaF_2_(185.96)/VO_2_(10)/CaF_2_(10)/VO_2_(10)/CaF_2_(86.94)	46.18%	41.89%	13.35%	46.29%	1.744

a *T*_vis,l_and *T*_vis,h_ denote the visible transmittance in the low- and high-temperature states, respectively; Δ*T*_sol_ and Δ*ε*_avg_ denote the solar transmittance modulation and average infrared emissivity modulation, respectively; *F* is the dimensionless composite evaluation score.

Overall, the comprehensive performance of the film system improved significantly as the number of layers increased. The spectral response of the three-layer structure is shown in Fig. 3(a). Although this structure achieves a solar modulation capability of 12.69% and an emissivity modulation capability of 45.21%, its visible light transmittance at low and high temperatures is only 45.46% and 41.71%, respectively, and its comprehensive evaluation value 
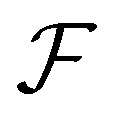
 is merely 1.705. This indicates that although a single D/M/D resonator possesses basic spectral control capabilities, it still faces significant limitations in terms of visible light transmittance.

When the structure was expanded to five layers, the performance of the film system improved significantly. The optimized five-layer structure achieved the highest visible light transmittance among all configurations in both the low-temperature state (51.06%) and the high-temperature state (46.97%), while maintaining a solar modulation capability of 12.28% and an emissivity modulation capability of 47.33%. Consequently, the comprehensive evaluation value 
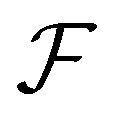
 increased to 1.781. As shown in Fig. 3(b), the five-layer structure maintains good transmission characteristics in the visible light band, while in the mid- and far-infrared bands, distinct differences in emissivity are observed under different temperature states. These results indicate that appropriately increasing the number of layers can introduce greater flexibility in interface reflection and phase control, thereby optimizing the interference transmission conditions in the visible light band while still maintaining strong solar radiation modulation and infrared emissivity control capabilities, demonstrating superior multi-objective co-optimization effects.

After further increasing the number of layers to seven, the structure's comprehensive evaluation value 
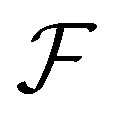
 remained at 1.781, essentially unchanged from the five-layer structure; its spectral response is shown in Fig. 3(c). The seven-layer structure exhibited a slight improvement in emissivity modulation capability, but its visible light transmittance decreased compared to the five-layer structure. This indicates that while adding more layers can enhance the control capability in certain wavelength bands, the overall benefits derived from this approach are becoming limited. The spectral curve of the nine-layer structure is shown in Fig. 3(d). Although the nine-layer structure achieved the highest solar modulation capability (13.35%), its visible light transmittance decreased significantly, resulting in a reduction of the comprehensive evaluation value 
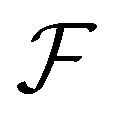
 to 1.744. This indicates that an excessive number of layers introduces additional reflection and absorption losses, which is detrimental to improving overall performance.

In summary, while increasing the number of layers can enhance the spectral tuning flexibility of multilayer films to some extent, the improvement in performance does not increase monotonically with the number of layers. Taking into account optical performance, structural complexity, and the feasibility of future fabrication, this study concludes that a five-layer CaF_2_/VO_2_ alternating symmetric multilayer structure represents the optimal solution for the current system and will serve as the focal point for subsequent spectral analysis and mechanistic discussions.

### 3.2 Explanation of the electric field mechanism

**Fig. 4:**
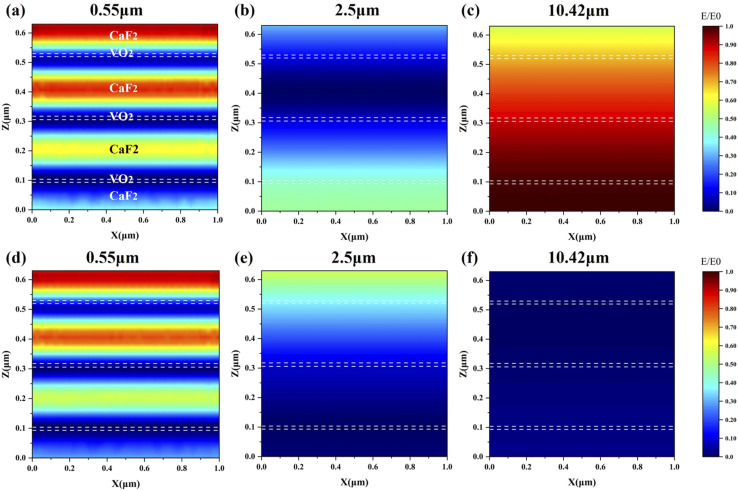
Normalized electric field distributions of the optimized structure at different wavelengths. (a–c) Electric field distributions at 0.55 µm, 2.5 µm, and 10.42 µm in the low-temperature state; (d–f) electric field distributions at 0.55 µm, 2.5 µm, and 10.42 µm in the high-temperature state. The white dashed lines indicate the interface positions between different film layers.

To reveal the spectral regulation mechanism of the optimized structure, three characteristic wavelengths, 0.55 µm (visible light band), 2.5 µm (solar band), and 10.42 µm (mid-infrared band), were selected to analyze the normalized electric field distributions of VO_2_ in the low-temperature and high-temperature states, respectively, as shown in Fig. 4. Specifically, Fig. 4(a–c) correspond to the electric field distributions at 0.55 µm, 2.5 µm, and 10.42 µm in the low-temperature state, while Fig. 4(d–f) correspond to the electric field distributions at the same wavelengths in the high-temperature state.

At a wavelength of *λ*_1_ = 0.55 µm in the visible light band, Fig. 4(a) and (d) show that the electric field distribution of VO_2_ remains highly consistent between the low-temperature and high-temperature states. The optical field exhibits a layered standing wave pattern along the thickness direction, with no significant field restructuring observed due to phase transitions. This indicates that the optimized multilayer structure is capable of maintaining a relatively stable visible light transmission channel through phase compensation in the dielectric layers and multi-interface interference. Consequently, this structure retains high visible light transmittance while simultaneously supporting the dynamic control function of VO_2_.

As the wavelength increases to *λ*_2_ = 2.5 µm, the electric field distributions in Fig. 4(b) and (e) begin to exhibit distinct temperature dependence. In the low-temperature state, the light field can still form a certain degree of coupled distribution within the film layers. In the high-temperature state, however, the distribution of the electric field along the thickness direction is reorganized, and the field strength in some regions is suppressed. This change indicates that, as the spectrum enters the near-infrared and mid-infrared transition regions, the changes in complex refractive index caused by the VO_2_ phase transition begin to significantly alter the phase-matching conditions and energy transfer pathways of the multilayer film. Consequently, a near-infrared transmittance difference between the high- and low-temperature states gradually emerges, further contributing to the structure's solar energy modulation capability.

At the mid-infrared characteristic wavelength *λ*_3_ = 10.42 µm, Fig. 4(c) and (f) show the most significant differences in phase transition response. In the low-temperature state, the electric field can penetrate sufficiently into the interior of the multilayer film, indicating that the insulating VO_2_ still maintains a certain degree of mid-infrared coupling with adjacent layers. However, when VO_2_ transitions to the high-temperature metallic state, the overall electric field strength within the film decreases markedly, and the propagation of mid-infrared electromagnetic waves within the structure is more strongly shielded. This pronounced difference in electric field distribution indicates that the VO_2_ phase transition not only alters the material's intrinsic optical response but also re-modulates the interference and coupling conditions within the multilayer film. It is evident that the modulation of the structure's infrared emissivity primarily stems from the synergistic interaction between the VO_2_ phase transition response and the interference effects within the multilayer film.

### 3.3 Angle-sensitivity analysis

**Fig. 5:**
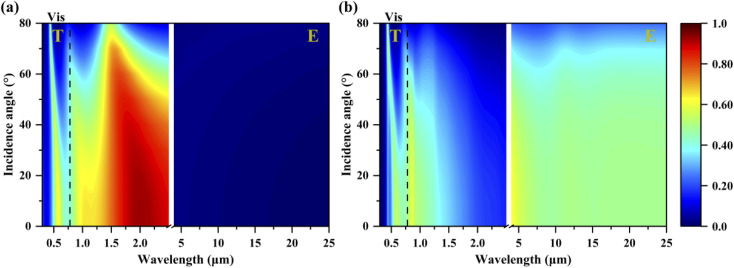
Spectral transmittance and emissivity distributions of the optimized structure at different incident angles. (a) Optimized structure in the low-temperature state; (b) optimized structure in the high-temperature state.

Since building windows are typically exposed to non-normal incident light over their service life, angular stability is a key indicator for evaluating the all-weather energy-saving performance of smart window films. To this end, this paper further calculates the spectral response of the optimized structure over an incident angle range of 0–80°, focusing primarily on transmittance in the solar spectrum and emissivity in the mid-infrared spectrum. The angular response results for the low-temperature and high-temperature states are shown in Fig. 5(a) and (b), respectively.

As shown in Fig. 5(a) (low-temperature results), within the 0–60° incidence range, the structure maintains strong transmittance characteristics in the visible and near-infrared solar bands, with no significant attenuation observed in the visible band. This indicates that the film can maintain its daylighting performance over a wide range of angles under low-temperature conditions. As the incident angle increases further, the transmittance in the solar bands gradually decreases. This is primarily attributed to the increased optical path length under oblique incidence, enhanced interfacial reflection, and a shift in the phase-matching conditions of the multilayer film. Meanwhile, the low-temperature state maintains a relatively low overall emissivity in the mid-infrared band, with minimal variation with angle, indicating stable thermal insulation properties under low-temperature conditions. Fig. 5(b) shows that, under high-temperature conditions, solar band transmittance is generally lower than that under low-temperature conditions, exhibiting a more pronounced blocking effect particularly in the near-infrared band. This indicates that the metallic state response formed after the VO_2_ phase transition can suppress the entry of solar thermal radiation into the interior over a wide range of angles. On the other hand, the high-temperature state maintains a high emissivity in the 5–25 µm mid-infrared band, and this emissivity does not degrade significantly over a wide range of incident angles, indicating that the structure can maintain strong thermal radiation emission capabilities under high-temperature conditions. This characteristic is beneficial for reducing indoor heat loads during summer or under high-temperature conditions.

Overall, the film maintains a temperature-selective response under different incident angles, featuring high transmittance in the low-temperature state and enhanced blocking and radiative regulation in the high-temperature state, demonstrating its good angular stability and practical potential for smart window applications.

### 3.4 Energy efficiency simulation

**Fig. 6:**
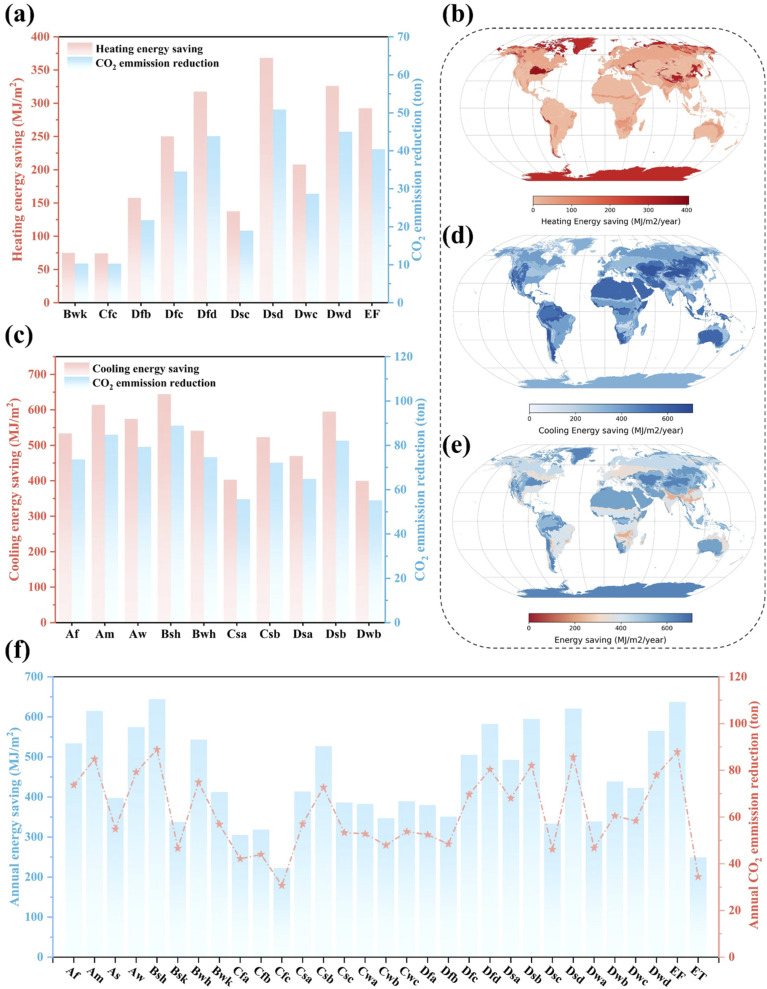
EnergyPlus-based building energy consumption simulation results. (a) Heating energy savings and corresponding CO_2_ emission reductions for the top 10 Köppen–Geiger climate zones. (b) Global distribution of heating energy savings. (c) Cooling energy savings and corresponding CO_2_ emission reductions for the top 10 Köppen–Geiger climate zones. (d) Global distribution of cooling energy savings. (e) Global distribution of total annual energy savings. (f) Total annual energy savings and corresponding CO_2_ emission reductions across 31 Köppen–Geiger climate zones.

To further evaluate the energy-saving potential of the designed smart window film in actual buildings, this study established a representative multi-story building model using EnergyPlus. By combining this model with Köppen-Geiger climate zones and representative meteorological data, annual energy consumption simulations were conducted across a range of climate zones, including tropical, arid, temperate, continental, and polar regions. The specific climate types corresponding to each climate code are listed in SI Table S6. The EnergyPlus model parameters are provided in SI Table S7, and the simulation results are shown in Fig. 6.

The results indicate that, for heating-dominated climate zones, energy-saving benefits are primarily observed in cold and high-latitude regions, as shown in Fig. 6(a) and (b). Under low-temperature conditions, VO_2_ maintains high solar transmittance and low mid-infrared emissivity, which facilitates the entry of winter solar radiation into indoor spaces while reducing long-wave thermal radiation losses, thereby lowering heating energy consumption. Therefore, this smart window is not only suitable for thermal insulation and cooling in hot regions but also serves to passively increase temperature and retain heat in cold regions.

Additionally, the optimized smart window film described in this paper demonstrates significant energy-saving advantages in cooling-dominated climate zones, with particularly notable effects in tropical, arid-hot, and high-solar-radiation regions, as shown in Fig. 6(c) and (d). Under high-temperature conditions, VO_2_ undergoes a phase transition that reduces solar transmittance and enhances the regulation of mid-infrared thermal radiation, thereby effectively reducing indoor solar heat gain and air conditioning cooling loads. Consequently, these regions also exhibit high CO_2_ emission reduction potential, indicating that this structure has significant application value in building environments with high cooling loads.

A further comparison of the annual energy-saving results across all climate zones, as shown in Fig. 6(e) and (f), reveals that this structure achieves positive energy savings in most climate zones. Among these, the tropical/hot semi-arid climate zone (BSh) achieved the highest energy savings, reaching 644.21 MJ per m^2^ year. The ice-cap climate zone (EF) and the extremely cold summer-dry climate zone (Dsd) also demonstrate exceptional performance, with annual energy savings of 636.99 and 620.88 MJ per m^2^ year, respectively. These results indicate that the energy-saving benefits of the designed smart window are not limited to hot or cold regions alone, but rather can make differentiated contributions to cooling and heating energy savings depending on different climatic conditions.

## 4 Conclusions

This study addresses the need for year-round energy conservation in buildings by establishing a smart optimization design framework for phase-change multilayer smart window films. It introduces DQN-based inverse design strategy to automatically optimize the layer structure and thickness parameters within a system of multiple candidate materials. The optimization results indicate that the alternating symmetric CaF_2_/VO_2_ multilayer structure exhibits excellent ultra-wideband synergistic control capabilities in both the solar and mid-to-far-infrared bands, with the five-layer CaF_2_/VO_2_/CaF_2_/VO_2_/CaF_2_ structure achieving the optimal balance between optical performance and structural complexity. This structure exhibits visible light transmittance values of 51.06% and 46.97% in the low- and high-temperature states, respectively, with a solar modulation capability of 12.28% and an average emissivity modulation capability of 47.33% in the mid-to-far-infrared range. Electric field distribution analysis reveals that the VO_2_ phase transition minimally disturbs the visible transmission channel while significantly reconfiguring phase matching and electromagnetic coupling states. Angular response results confirm that this structure maintains stable temperature-selective spectral characteristics over a wide range of incident angles. Building energy consumption simulations based on EnergyPlus further demonstrate that this smart window possesses energy-saving potential across different climate zones. The highest annual energy savings are observed in the BSh climate zone (644.21 MJ per m^2^ per year), followed by the EF (636.99 MJ per m^2^ per year) and Dsd (620.88 MJ per m^2^ per year) zones. These results indicate that the multilayer smart window film proposed in this study can achieve climate-adaptive regulation between cooling and heating demands, providing an effective solution for the design of ultra-broadband radiation-regulating smart windows aimed at year-round building energy conservation.

## Conflicts of interest

There are no conflicts to declare.

## Supplementary Material

RA-OLF-D6RA04939G-s001

## Data Availability

The data are available from the corresponding author on reasonable request. Ref. 45–62 are cited in the supplementary information (SI). Supplementary information: additional details on material optical constants, DQN optimization and training parameters, material screening and convergence results, comparison with representative smart-window systems, and the EnergyPlus building energy simulation setup. See DOI: https://doi.org/10.1039/d6ra04939g.
