# Supplementary material for: Smart window films with ultra-broadband radiative regulation for all-season building energy saving
Source: RSC Adv. 2026 Aug 18;16(41):48587–99. doi: 10.1039/d6ra04939g (PMC13482964; doi:10.1039/d6ra04939g)
Supplement: Supplementary file 1 [file RA-016-D6RA04939G-s001.pdf]

Supplementary Information (SI) for RSC Advances.
This journal is © The Royal Society of Chemistry 2026

Smart Window Films with Ultra-Broadband Radiative Regulation for All-Season Building Energy Saving

Wenjuan Jiang, Haining Ji*, Juantao Zhang, Xiyu Wu,

Runteng Luo, Zhi Zeng, Tianjian Xiao

School of Physics and Optoelectronics, Xiangtan University, Xiangtan, Hunan 411105, P. R. China

sdytjhn@126.com

The detailed training parameters for DQN optimization are summarized in Table S2. At the beginning of each episode, 50 candidate states are randomly generated within the corresponding design space, and the state with the highest composite evaluation score is selected as the initial state for that episode. In the first stage, the material code is randomly selected from the set of candidate materials, and the two independent layer thickness parameters are each independently sampled from a uniform distribution within the range of 0.01–0.35 μm; in the second stage, the material system is fixed, and the independent layer thickness parameters are similarly sampled uniformly from the aforementioned range. Each independent training run executes up to 100,000 environment interaction steps, and the maximum length of a single episode is 10,000 steps. When a single episode reaches its maximum number of steps, the environment is reinitialized and the next episode begins; the entire training process terminates after a cumulative total of 100,000 steps is reached. If the composite evaluation metric does not improve for 500 consecutive steps, the model's state is reset to continue the search, rather than terminating training immediately.

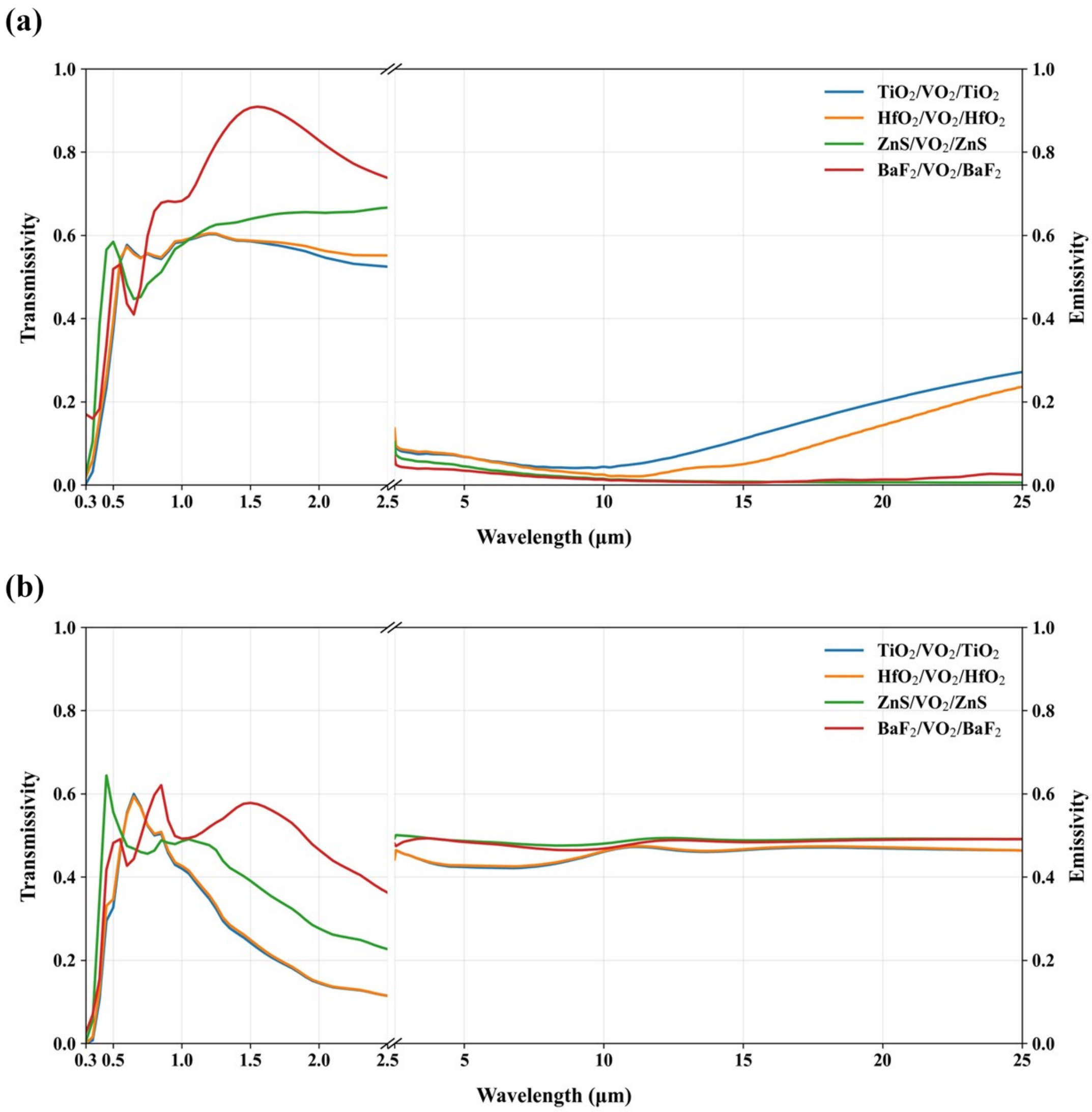

Figure S1. Spectral performance of representative optimized three-layer candidate structures at (a) low temperature and (b) high temperature, including solar-band transmittance and mid-to-far-infrared emissivity.

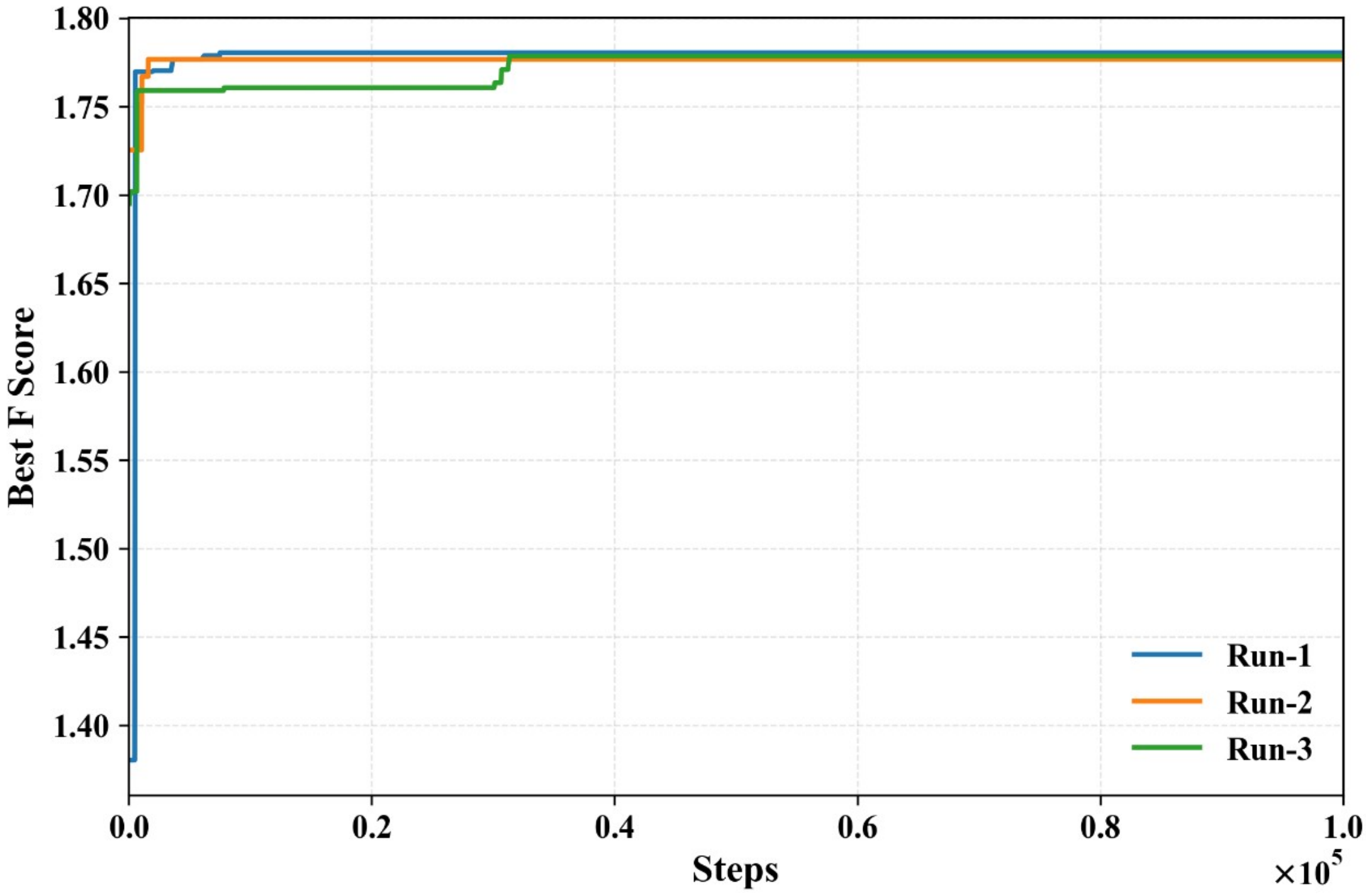

Figure S2. Convergence curves of the best-so-far composite evaluation score from three independent DQN optimization runs for the five-layer $CaF_2/VO_2/CaF_2/VO_2/CaF_2$ structure.

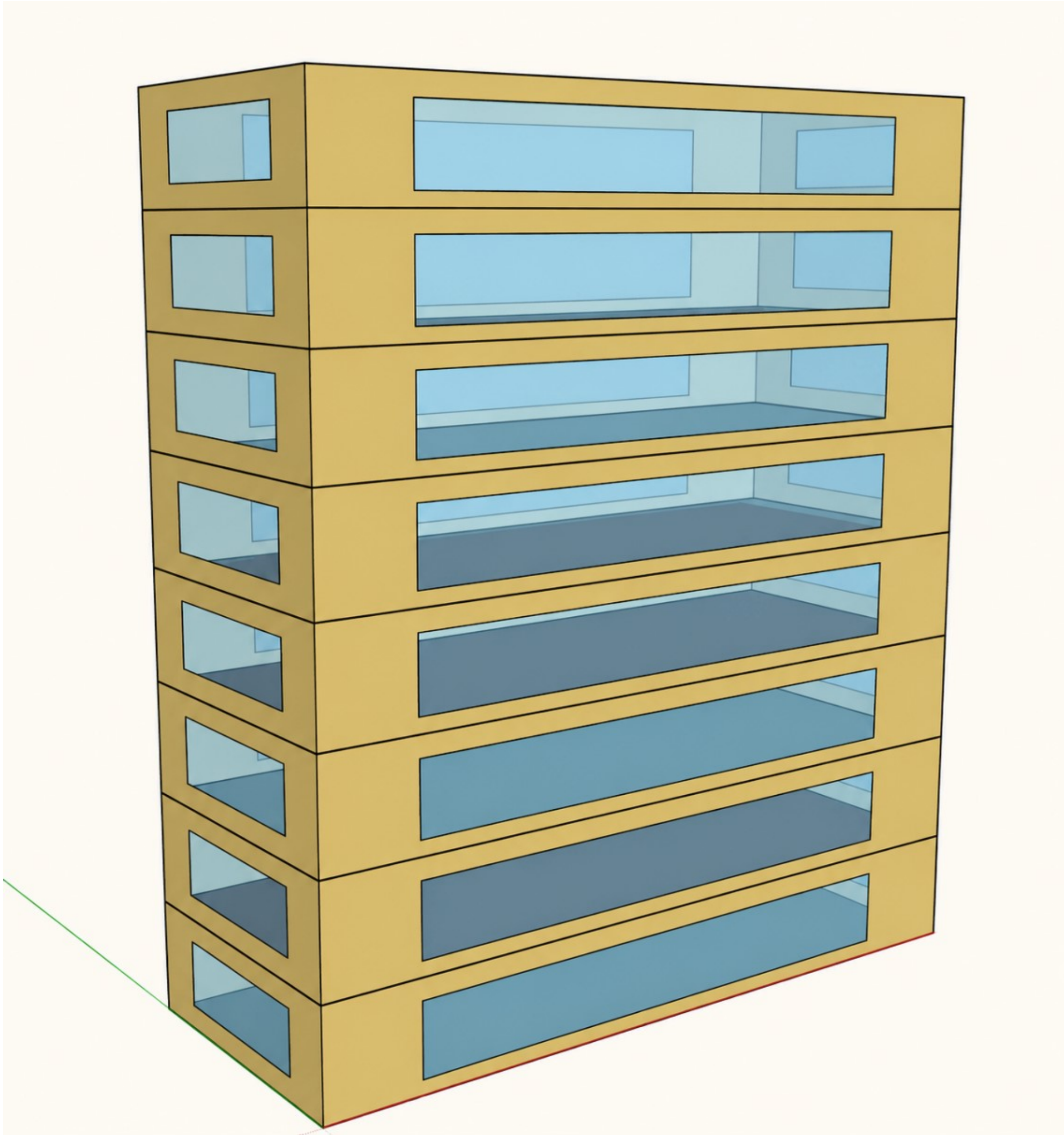

Figure S3. Building model used in the EnergyPlus building energy consumption simulation.

Table S1. Material codes and sources of optical constants used in this work.

The optical constants of all candidate materials used in the optical simulations were taken from previously reported data, as summarized in Table S1.

**Material Coding**	**Material Name**	**Reference**	**Material Coding**	**Material Name**	**Reference**
0	Ag	Yang et al.[45]	8	HfO_2	Bright et al.[46]
1	Al	Rakic et al.[47]	9	Si	Franta et al.[48]
2	AlN	Kischkat et al.[49]	10	Si_3N_4	Luke et al.[50]
3	Al_2O_3	Franta et al.[51]	11	SiO_2	Franta et al.[52]
4	BaF_2	Querry[53]	12	TiO_2	Franta et al.[51]
5	CaF_2	Li[54]	13	VO_2 (HT/LT)	Wan et al. [55]
6	GaAs	Adachi[56]	14	ZnS	Querry[53]
7	GeAs	Mead[57]	15	ZnSe	Querry[53]

Table S2. Comparison of representative optimized candidate structures and their optical performance

Structure (thickness / nm)	$T_{vis,l}$	$T_{vis,h}$	ΔT_{sol}	$\Delta\varepsilon_{avg}$	F
TiO_2(40.47) / VO_2(49.88) / TiO_2(40.47)	51.80%	47.19%	9.19%	33.31%	1.549
HfO_2(41.47) / VO_2(48.88) / HfO_2(41.47)	52.41%	47.80%	9.17%	36.88%	1.584
ZnS(31.47) / VO_2(28.88) / ZnS(31.47)	52.56%	50.76%	6.17%	47.11%	1.596
BaF_2(250.45) / VO_2(26.69) / BaF_2(250.45)	49.00%	46.64%	9.26%	46.58%	1.680
**CaF_2(282.33) / VO_2(39.71) / CaF_2(282.33) [a]**	**45.46%**	**41.71%**	**12.69%**	**45.21%**	**1.705**

[a] The CaF_2/VO_2/CaF_2 structure in the final row was selected for subsequent structural expansion and further analysis

Table S3. DQN hyperparameter configuration

Parameter	Setting
Independent thickness variables	3
Thickness range	0.01–0.35 μm
Thickness increments	±0.001, ±0.005, ±0.010 μm
Hidden layers	2 × 128
Activation function	Tanh
Replay-memory capacity	15,000
Mini-batch size	64
Optimizer	Adam
Learning rate	5×10^{-4}
Discount factor	0.99
Loss function	Smooth L1
Warm-up period	15,000 steps
Online-network update	Every 4 steps
Target-network update	Every 2,000 steps
Maximum training length	100,000 steps
Maximum episode length	10,000 steps
Initial/final ε	1.0/0.05
ε-decay period	First 90% of training
Number of independent runs	3
Adam beta parameters	β_1=0.9, β_2=0.999 (PyTorch defaults)
Gradient clipping	Global L_2-norm clipping, maximum norm = 10.0
Reward clipping	[−10,10]

Table S4. Optimized structures and optical performance obtained from three independent DQN training runs.

Run	Optimized structure with layer thicknesses (nm)	$T_{vis,l}$ (%)	$T_{vis,h}$ (%)	ΔT_{sol} (%)	Δε (%)	Best score
1	CaF_2(76.95)/VO_2(14.99)/CaF_2(196.61)/VO_2(14.99)/CaF_2(76.95)	50.47	47.27	11.85	47.80	1.7735
2	CaF_2(76.20)/VO_2(15.38)/CaF_2(207.46)/VO_2(15.38)/CaF_2(76.20)	50.86	47.11	12.09	47.89	1.7816
**3**	**CaF_2(86.51)/VO_2(16.34)/CaF_2(207.32)/VO_2(16.34)/CaF_2(86.51)** [b]	**51.06**	**46.97**	**12.28**	**47.33**	**1.7810**
Mean ± SD	—	50.80 ± 0.30	47.12 ± 0.15	12.07 ± 0.22	47.68 ± 0.30	1.7787 ± 0.0045

[b] The optimized structure obtained in Run 3 corresponds to the design reported in the main text and was used for subsequent performance analyses.

Table S5. Comparison with representative VO_2-based smart windows and commercial low-E glazing.

Structure	Type	Tvis	ΔTsol	Δε	Spectral range for Δε	Reference
VO_2/PMMA/ITO/glass/ITO	Experimental	27	9.3	40	LWIR	Wang et al., Science (2021)[58]
Porous VO_2/ZnSe/ITO/glass	Experimental and simulation	41	N.R.	≥40	LWIR	Bhupathi et al., Nanophotonics (2024)[59]
Patterned VO_2/CaF_2/Ag	Numerical design	50.4	13.9	49	2.5–25 μm	Ou et al., Solar Energy (2024)[60]
VO_2/TiO_2/ITO	Experimental and simulation	38.5	8.5	42.5	LWIR	Keum et al., Materials Today Physics (2025)[61]
Guardian SunGuard SNX 60+ on Clear	Commercial static low-E glazing	60	N.A.	N.A.	Static low-E	Guardian Glass product data[62]
**CaF_2/VO_2/CaF_2/VO_2/CaF_2**	**Numerical design**	**51.06/ 46.97**	**12.28**	**47.33**	**2.5–25 μm**	**This work**

Note: N.R., not reported; N.A., not applicable.

Table S6. Köppen-Geiger climate classification codes used in this study

Code	Climate type	General description
**Af**	Tropical rainforest climate	Tropical climate with abundant rainfall throughout the year and no dry season.
**Am**	Tropical monsoon climate	Tropical climate with a short dry season and strong monsoon-controlled rainfall.
**As**	Tropical savanna climate with dry summer	Tropical savanna climate in which the driest season occurs in summer.
**Aw**	Tropical savanna climate with dry winter	Tropical savanna climate with a distinct dry winter and wet summer.
**BSh**	Hot semi-arid climate	Arid climate with limited precipitation and relatively high annual temperature.
**BSk**	Cold semi-arid climate	Semi-arid climate with lower annual temperature than hot semi-arid regions.
**BWh**	Hot desert climate	Desert climate characterized by very low precipitation and high temperature.
**BWk**	Cold desert climate	Desert climate characterized by very low precipitation and lower annual temperature.
**Cfa**	Humid subtropical climate	Temperate climate with hot summers and precipitation throughout the year.
**Cfb**	Temperate oceanic climate	Temperate climate with mild summers and precipitation throughout the year.
**Cfc**	Subpolar oceanic climate	Cool oceanic climate with short, cool summers and year-round moisture.
**Csa**	Hot-summer Mediterranean climate	Temperate climate with hot, dry summers and wet winters.
**Csb**	Warm-summer Mediterranean climate	Temperate climate with warm, dry summers and wet winters.
**Csc**	Cold-summer Mediterranean climate	Cool Mediterranean climate with dry summers and wet winters.
**Cwa**	Dry-winter humid subtropical climate	Temperate climate with hot summers, wet summers, and dry winters.
**Cwb**	Subtropical highland climate with dry winter	Highland temperate climate with warm summers and dry winters.
**Cwc**	Cold subtropical highland climate with dry winter	Highland temperate climate with cool summers and dry winters.
**Dfa**	Hot-summer humid continental climate	Continental climate with hot summers, cold winters, and year-round precipitation.

Code	Climate type	General description
**Dfb**	Warm-summer humid continental climate	Continental climate with warm summers, cold winters, and year-round precipitation.
**Dfc**	Subarctic climate	Cold continental climate with short, cool summers and long cold winters.
**Dfd**	Extremely cold subarctic climate	Subarctic climate with extremely cold winters.
**Dsa**	Hot-summer dry-summer continental climate	Continental climate with hot, dry summers and cold winters.
**Dsb**	Warm-summer dry-summer continental climate	Continental climate with warm, dry summers and cold winters.
**Dsc**	Dry-summer subarctic climate	Subarctic climate with cool, dry summers and long cold winters.
**Dsd**	Extremely cold dry-summer subarctic climate	Subarctic climate with dry summers and extremely cold winters.
**Dwa**	Hot-summer dry-winter continental climate	Continental climate with hot summers, cold dry winters, and wet summers.
**Dwb**	Warm-summer dry-winter continental climate	Continental climate with warm summers, cold dry winters, and wet summers.
**Dwc**	Dry-winter subarctic climate	Subarctic climate with short cool summers and dry winters.
**Dwd**	Extremely cold dry-winter subarctic climate	Subarctic climate with dry winters and extremely cold winter temperatures.
**EF**	Ice-cap climate	Polar climate in which the warmest month remains below 0 °C.
**ET**	Tundra climate	Polar climate with a warmest-month temperature between 0 °C and 10 °C.

Note: The first letter denotes the major climate group: A = tropical, B = arid, C = temperate, D = continental, and E = polar. Subsequent letters describe seasonal precipitation and thermal characteristics.

Table S7. Key parameters of the EnergyPlus building model.

Parameter	Value
Building structure	8-story building model
Total wall area	2267.8 m^2
Total glass area	1003.5 m^2
Roof area	281.4 m^2
Floor area	321.60 m^2 per floor
HVAC system type	Ideal air-conditioning system
Heating supply air temperature	50 °C
Cooling supply air temperature	13 °C
Supply air humidity ratio	0.0156 for heating; 0.0077 for cooling

References

(45) Yang, H.; D'Archangel, J.; Sundheimer, M.; Tucker, E.; Boreman, G.; Raschke, M. Optical dielectric function of silver. *Phys. Rev. B* **2015**, *91* (23), 11. DOI: 10.1103/PhysRevB.91.235137.

(46) Bright, T. J.; Watjen, J. I.; Zhang, Z. M.; Muratore, C.; Voevodin, A. A. Optical properties of HfO2 thin films deposited by magnetron sputtering: From the visible to the far-infrared. *Thin Solid Films* **2012**, *520* (22), 6793–6802. DOI: https://doi.org/10.1016/j.tsf.2012.07.037.

(47) Rakić, A. D.; Djurišić, A. B.; Elazar, J. M.; Majewski, M. L. Optical properties of metallic films for vertical-cavity optoelectronic devices. *Appl. Opt.* **1998**, *37* (22), 5271–5283. DOI: 10.1364/AO.37.005271.

(48) Franta, D.; Nečas, D.; Zajíčková, L.; Ohlídal, I.; Stuchlík, J. Advanced modeling for optical characterization of amorphous hydrogenated silicon films. *Thin Solid Films* **2013**, *541*, 12–16. DOI: https://doi.org/10.1016/j.tsf.2013.04.129.

(49) Kischkat, J.; Peters, S.; Gruska, B.; Semtsiv, M.; Chashnikova, M.; Klinkmüller, M.; Fedosenko, O.; Machulik, S.; Aleksandrova, A.; Monastyrskyi, G.; et al. Mid-infrared optical properties of thin films of aluminum oxide, titanium dioxide, silicon dioxide, aluminum nitride, and silicon nitride. *Appl. Opt.* **2012**, *51* (28), 6789–6798. DOI: 10.1364/AO.51.006789.

(50) Luke, K.; Okawachi, Y.; Lamont, M. R. E.; Gaeta, A. L.; Lipson, M. Broadband mid-infrared frequency comb generation in a Si3N4 microresonator. In *2015 Conference on Lasers and Electro-Optics (CLEO)*, 10–15 May 2015, 2015; pp 1–2.

(51) Franta, D.; Nečas, D.; Ohlídal, I.; Giglia, A. Dispersion model for optical thin films applicable in wide spectral range. In Proceedings of SPIE, Bellingham, WA, USA; 2015.

(52) Franta, D.; Nečas, D.; Ohlídal, I.; Giglia, A. Optical characterization of SiO2 thin films using universal dispersion model over wide spectral range. In Optical Micro- and Nanometrology VI, Brussels, Belgium; 2016.

(53) Bell, R. J.; Alexander, R. W.; Newquist, L. A.; Van Diver, R.; Ordal, M. A. Optical Constants of Minerals and Other Materials from the Millimeter to the Ultraviolet. **1988**.

(54) Li, H. H. Refractive index of alkaline earth halides and its wavelength and temperature derivatives. *Journal of Physical and Chemical Reference Data* **1980**, *9* (1), 161–290. DOI: 10.1063/1.555616 (accessed 5/25/2026).

(55) Wan, C.; Zhang, Z.; Woolf, D.; Hessel, C. M.; Rensberg, J.; Hensley, J. M.; Xiao, Y.; Shahsafi, A.; Salman, J.; Richter, S.; et al. On the Optical Properties of Thin-Film Vanadium Dioxide from the Visible to the Far Infrared. *Annalen der Physik* **2019**, *531* (10), 1900188. DOI: https://doi.org/10.1002/andp.201900188 (accessed 2026/07/25).

(56) Adachi, S. Optical dispersion relations for GaP, GaAs, GaSb, InP, InAs, InSb, AlxGa1−xAs, and In1−xGaxAsyP1−y. *Journal of Applied Physics* **1989**, *66* (12), 6030–6040. DOI: 10.1063/1.343580 (accessed 5/25/2026).

(57) Mead, D. G. Long wavelength study of semiconducting germanium arsenide, GeAs. *Infrared Physics* **1982**, *22* (4), 209–213. DOI: https://doi.org/10.1016/0020-0891(82)90045-8.

(58) Wang, S.; Jiang, T.; Meng, Y.; Yang, R.; Tan, G.; Long, Y. Scalable thermochromic smart windows with passive radiative cooling regulation. *Science* **2021**, *374* (6574), 1501–1504. DOI: doi:10.1126/science.abg0291.

(59) Bhupathi, S.; Wang, S.; Wang, G.; Long, Y. Porous vanadium dioxide thin film-based Fabry−Perot cavity system for radiative cooling regulating thermochromic windows: experimental and simulation studies. *Nanophotonics* **2024**, *13* (5), 711–723. DOI: https://doi.org/10.1515/nanoph-2023-0716 (accessed 2026/07/24).

(60) Ou, Y.; Ji, H.; Wang, Y.; Liu, B.; Chen, Y.; Tao, J.; Huang, Y.; Wang, J. Photothermal synergistic modulation of patterned VO2-Based composite films for smart windows. *Sol. Energy* **2024**, *279*, 112808. DOI: https://doi.org/10.1016/j.solener.2024.112808.

(61) Keum, J.; Choi, J.; Kim, S.; Kang, G.; Lee, B.; Lee, M. J.; Kim, W. Innovative dual-band energy-efficient smart windows using VO2(M)-Based Fabry-Pérot structures for solar and radiative cooling modulation. *Materials Today Physics* **2025**, *52*, 101665. DOI: https://doi.org/10.1016/j.mtphys.2025.101665.
(62) Guardian, G. SunGuard SNX 60+ on Clear. *Guardian Glass*. (accessed 2026/07/25).
